# Essential Defects of the Theatre and Cinema

**Published:** 1917-08-11

**Authors:** 


### WAR MATINEES AND THE PUBLIC HEALTH.

### Essential Defects of the Theatre and Cinema.

Amongst humorous writings are several which point the moral that the human being is so constituted as willingly to undergo extraordinary inconvenience, discomfort, and fatigue in the pursuit of pleasure. This characteristic is, in part at least, the theme of "Three Men in a Boat" and "The Caravaners," being most amusingly treated in the latter as an especially British trait seen through the eyes of a German officer. There is also the delightful story of "a pleasure exertion" by an American authoress, Marietta Holley. Recent experiences at matinée theatrical performances in London have brought home to us the fact that, in the pursuit of amusement, people will readily breathe for hours close and fetid air; will incur the penalties of headache, ennui, and minor indisposition; and will run the risk of more serious illness. The matinée performance at theatre, music-hall, and picture-palace has become, for reasons sufficiently well known, increasingly popular; a few months ago it seemed even possible that such places of entertainment in London might for a time have to be closed in the evening. Theatres and halls are now often crowded by one audience during the daylight hours of the afternoon and, after a brief interval, by another in the evening, the evils attendant upon lack of ventilation being thus intensified.

The minor effects which follow upon a visit to a crowded and stuffy theatre are well known; only too often, however, the catarrh which follows is attributed to a draught or to "coming out into the cold air," when it should be attributed to infection or to the effect of impure air upon the mucous membrane of the naso-pharynx. Subtle and more serious are the dangers of exposure to the infections of the group of diseases the elusive organisms of which lurk in the throat of the carrier, often himself in apparent good health. In this group may be included influenza, diphtheria, cerebro-spinal fever, and, possibly, pneumonia and acute anterior poliomyelitis; to it are allied such highly contagious diseases as scarlet fever, whooping-cough, and measles. It is not from sheer perversity that medical officers of schools seek to keep boys and girls away from places of public assembly for a time before their return to school. A fresh and increasing importance has come to be attached to the influence of contagion in the spread of many diseases now known to be infective.

The essential defect of the theatre and cinema from the public health point of view is inefficiency of ventilation. We are well aware that the question of the hygienic well-being of a crowded audience is not one wholly of ventilation, and we know the difficulties of the subject. The crux of the matter of ventilation is to secure without draught (the elderly theatre-goer's bugbear!) a sufficient change of air to maintain a reasonable standard of purity; this means, for large numbers of persons closely seated on the floor-space, a surprisingly large bulk of fresh air—air, moreover, which should be warmed in cold weather. The degree of impurity at which air should be deemed unfit for respiration can only be arbitrarily decided; nor is the precise nature of the dangerous impurity certain. Recent researches tend to show that stagnation of air, allowing of the accumulation of products of respiration about the person and between the layers of the clothing, is a factor of considerable importance. To this cause may be in part due the well-known unsuitability of churches for use as emergency hospitals, the air of churches being notably stagnant. The science of ventilation has been applied with great success in modern shipbuilding, in which the conditions are often such that forced ventilation only is applicable. Efficient forced ventilation in the case of such a building as a theatre leads inevitably to draught; it has also been found that complicated and costly schemes of artificial ventilation have resulted in a peculiar devitalisation of air, and have rendered it inexplicably depressing to the healthy and unsuitable for the sick. "Natural" ventilation cannot be imitated, and an "open-air" condition is the ideal.

The ventilation of the theatre and the cinema remains, it must be allowed, an unsolved problem. The essential construction of such a building involves the accommodation of large numbers of people for a lengthy period in the absence of daylight, provision having to be made in the case of a matinée performance to exclude the entrance of light by any existing windows. Natural ventilation is, under these circumstances, practically impossible, especially as it is also desirable to shut out noises from without. In many cases means are provided for the exit of air through the ceiling of the auditorium and the roof of the stage, a "sliding-roof" has even been fitted to the former: to see the stars may give a sense of coolness to an audience, but, in the absence of means for the ingress of air below and at the back of the galleries, the actual escape of vitiated air through such a roof-opening is in still weather small, whilst by day and in wet weather it must be kept closed. The substitution of electric light for gas light has not been an unmixed blessing. The burning of gas consumed a large amount of oxygen, but the heat generated caused movement of air and greatly increased the efficiency of the ceiling or roof outlet.

The attraction of the "place of amusement," tax or no tax, was never greater than it is to-day, and the cinema has come to stay, banal though its productions have hitherto been. The risk to the public health of assembly in these places is undeniable. Architects and managers have it in their power to diminish these risks by improved ventilation and by the adoption of means of disinfection. The potent disinfectant action of sunlight being absent, and the methods of true disinfection being inapplicable, reliance should be placed on an extreme degree of cleanliness. Vaporised deodorants might be applied, especially between two consecutive performances, but their usefulness must not be over-estimated. Thorough cleansing by every available means is all-important, and no less necessary for the safety of the audience than a fireproof curtain is a vacuum cleaning outfit. This should be available throughout the auditorium, and should be of the kind which removes by powerful suction through pipes to the outer air every particle of dust from ledges, seating, carpets, etc. Its use is imperatively needed, and should be compulsory. As regards ventilation, we believe that the most promising method is an exhaust system by which natural inlets are left free of heavy curtains and other obstructions, while movement of air from the building is ensured by the use of silent-running exhaust fans over the heads of the audience.

